# Clinical characteristics, sepsis interventions and outcomes in the obese patients with septic shock: an international multicenter cohort study

**DOI:** 10.1186/cc12680

**Published:** 2013-04-17

**Authors:** Yaseen M Arabi, Saqib I Dara, Hani M Tamim, Asgar H Rishu, Abderrezak Bouchama, Mohammad K Khedr, Daniel Feinstein, Joseph E Parrillo, Kenneth E Wood, Sean P Keenan, Sergio Zanotti, Greg Martinka, Aseem Kumar, Anand Kumar

**Affiliations:** 1 Intensive Care Department, King Abdulaziz Medical City, Riyadh11426, Saudi Arabia; 2 Department of Epidemiology and Biostatistics, King Abdullah International Medical Research Center, Riyadh11426, Saudi Arabia; 3 Department of Experimental Medicine, King Abdullah International Medical Research Center, Riyadh 11426, Saudi Arabia; 4 Critical Care Medicine, Moses H. Cone Memorial Hospital, Greensboro, NC 27403, USA; 5 Heart and Vascular Hospital, UMDNJ, Hackensack University Medical Center, 30 Prospect Avenue, Hackensack, New Jersey 07601, USA; 6 Geisinger Medical Center, 100 North Academy Ave Danville, PA 17822-0111, USA; 7 Royal Columbian Hospital, 330 E Columbia St New Westminster, BC Canada V3L 3W7; 8 Cooper Hospital/University Medical Center,, 1 Cooper Plaza, Camden NJ, USA 08103; 9 Richmond Hospital, 7000 Westminster Highway, Richmond, B.C. V6X 1A2 Canada; 10 Laurentian University, 935 Ramsey Lake Rd. Sudbury, Ontario Canada P3E 2C6; 11 Section of Critical Care Medicine and Section of Infectious Diseases, Health Sciences Center and St. Boniface Hospital, University of Manitoba, Canada. 700 William Ave, Winnipeg, MB Canada R3P-1R9

## Abstract

**Introduction:**

Data are sparse as to whether obesity influences the risk of death in critically ill patients with septic shock. We sought to examine the possible impact of obesity, as assessed by body mass index (BMI), on hospital mortality in septic shock patients.

**Methods:**

We performed a nested cohort study within a retrospective database of patients with septic shock conducted in 28 medical centers in Canada, United States and Saudi Arabia between 1996 and 2008. Patients were classified according to the World Health Organization criteria for BMI. Multivariate logistic regression analysis was performed to evaluate the association between obesity and hospital mortality.

**Results:**

Of the 8,670 patients with septic shock, 2,882 (33.2%) had height and weight data recorded at ICU admission and constituted the study group. Obese patients were more likely to have skin and soft tissue infections and less likely to have pneumonia with predominantly Gram-positive microorganisms. Crystalloid and colloid resuscitation fluids in the first six hours were given at significantly lower volumes per kg in the obese and very obese patients compared to underweight and normal weight patients (for crystalloids: 55.0 ± 40.1 ml/kg for underweight, 43.2 ± 33.4 for normal BMI, 37.1 ± 30.8 for obese and 27.7 ± 22.0 for very obese). Antimicrobial doses per kg were also different among BMI groups. Crude analysis showed that obese and very obese patients had lower hospital mortality compared to normal weight patients (odds ratio (OR) 0.80, 95% confidence interval (CI) 0.66 to 0.97 for obese and OR 0.61, 95% CI 0.44 to 0.85 for very obese patients). After adjusting for baseline characteristics and sepsis interventions, the association became non-significant (OR 0.80, 95% CI 0.62 to 1.02 for obese and OR 0.69, 95% CI 0.45 to 1.04 for very obese).

**Conclusions:**

The obesity paradox (lower mortality in the obese) documented in other populations is also observed in septic shock. This may be related in part to differences in patient characteristics. However, the true paradox may lie in the variations in the sepsis interventions, such as the administration of resuscitation fluids and antimicrobial therapy. Considering the obesity epidemic and its impact on critical care, further studies are warranted to examine whether a weight-based approach to common therapeutic interventions in septic shock influences outcome.

## Introduction

Obesity is a fast growing epidemic worldwide and is closely associated with morbid conditions including diabetes, cardiovascular and respiratory diseases as well as cancer [1]. Approximately 65% of the United States population is overweight and 30% are obese. Obesity is increasingly a major health hazard in many developed and developing nations as well [2-4]. As a result, the proportion of obese patients admitted to hospitals is steadily increasing with an estimated cost that exceeds 5% of the national health expenditure in the US [2,3]. Therefore, obesity became the target for national-level endeavors as evidenced by the most recent release of the Institute of Medicine recommendations to 'Solve the Weight of the Nation' [5].

The prevalence of obese patients admitted to ICUs is also rising rapidly and poses complex challenges [6,7]. However, intriguingly, despite the increased morbidity and the difficulty of administering standard care, data on outcome, although conflicting, are showing predominantly either equal or lower mortality in obese than in normal weight critically ill patients, while only a few reported higher mortality [8,9]. A comparable phenomenon was also observed in obese patients with heart failure and is referred to as the 'obesity paradox' [10]. Although an explanation of this paradox is not immediately clear, most of the studies have included either a heterogeneous population of obese critically ill patients or have failed to adjust for major confounding factors, such as sepsis interventions [11,12]. The influence of obesity on specific ICU populations, such as patients with sepsis, has been the subject of much speculation but very few clinical data exist on this topic.

Sepsis is a major cause of morbidity and mortality worldwide and a leading admission diagnosis to ICUs [13] with substantial cost and considerable long-term health-related consequences [14-16]. Data on the impact of obesity on septic shock are primarily based on experimental and small clinical studies [17,18].

Therefore, we sought to examine the association of obesity, assessed by body mass index (BMI), and hospital mortality in patients admitted with septic shock. We further assessed the differences in clinical and microbiologic features as well as septic shock-related interventions in association with obesity.

## Materials and methods

### Subjects and setting

This was a retrospective cohort study from a large database of patients admitted with septic shock to the ICUs in 28 medical centers in Canada, United States, and Saudi Arabia by the Cooperative Antimicrobial Therapy of Septic Shock (CATSS) Database Research Group between 1996 and 2008. The study protocol was approved by the Instititutional Review Board of the University of Manitoba, Winnipeg, Canada; the University of Toronto, Toronto, Canada; McGill University, Montreal, Canada; the University of British Columbia, Vancouver, Canada; Rush University, Chicago, IL, USA; Brandon Hospital, Brandon, Canada; St. Agnes Hospital, Baltimore, MD, USA; Harper Hospital, Detroit, MI, USA; Northern Medical School, Laurentian University, Sudbury, Canada; the University of Calgary, Calgary, Canada; the University of Wisconsin, Madison, WI, USA; Cooper Hospital/University Medical Center, Camden, NJ, USA; Vancouver Island Regional Health Authority, Victoria, Canada; Hospital Maissoneuve-Rosemont, Montreal, Canada; and King Saud Bin Abdulaziz University for Health Sciences, Riyadh, Saudi Arabia. Informed consent was waived.

The original study included adult patients with septic shock defined according to the 1992 American College of Chest Physicians/Society of Critical Care Medicine guidelines [19]. The details regarding the full definitions used in the study have already been described [20,21]. Patients with weight and height documented in the medical records on admission were included in this study.

### Definition of obesity

Obesity was defined using the World Health Organization (WHO) criteria according to BMI (calculated as weight in kilograms divided by the square of the height in meters (kg/m^2^)) [22]. Patients were classified as underweight (BMI < 18.50 kg/m^2^), normal weight (BMI = 18.50 to 24.99 kg/m^2^), overweight (BMI = 25.0 to 29.99 kg/m^2^), obese (BMI = 30.0 to 39.99 kg/m^2^) or very obese (BMI > 40 kg/m^2^).

### Clinical characteristics and sepsis interventions

At baseline the following data were collected: age, gender, use of mechanical ventilation, Acute Physiology and Chronic Health Evaluation (APACHE) II score [23], admission physiologic data and comorbid conditions (immunosuppressive disorders, liver failure, heart failure, chronic obstructive pulmonary disease (COPD), chronic renal failure and medication- and insulin-dependent diabetes, alcohol abuse, elective and emergency surgery), laboratory data, organ failure indicators on admission [see Additional file 1, Appendix A], source of infection (community versus nosocomial infection), the presence of bacteremia, site of infection and microbiological data. We calculated creatinine clearance using the modification of diet in renal disease (MDRD) [24] and Cockcroft-Gault equations [25] [see Additional file 1, Appendix B]; the former was used in the multivariate analysis as it has been shown to perform better than Cockcroft-Gault equation in patients with extreme weights [26]. We calculated ideal and adjusted body weights and dosing weights for antimicrobials as recommended [6,27] [see Additional file 1, Appendix B].

We documented the following data regarding sepsis interventions: the volume of crystalloids, colloids, and blood products administered during the first six hours of septic shock (in ml and in ml/kg of actual body weight). We also recorded the selection and doses of vasopressors, use of activated protein C and low-dose steroids. The following data were collected about antimicrobial therapy: appropriateness, delay in hours from the onset of hypotension and the use of single versus combination antimicrobial therapy. Detailed definitions of these variables have already been described [20,21,28]. We also documented the type and dose (total and dose per kg of dosing body weight) of antimicrobials administered on the first day.

### Outcomes

Hospital mortality was the primary outcome. Secondary outcomes were ICU mortality and ICU and hospital length of stay (LOS).

### Statistical analysis

Continuous variables are reported as means with standard deviations (SD) and categorical variables as absolute and relative frequencies. Analysis of variance (ANOVA) and chi-square tests for comparison among groups were used as appropriate.

To study the association between obesity and hospital mortality, we carried out multivariate logistic regression analyses with the normal weight group as reference. We assessed the association of different BMI groups and mortality in crude and multivariate models adjusting for covariates that were selected based on clinical relevance or statistical criteria (*P *value of 0.25 for inclusion and *P *value of 0.05 for retaining in the model). We first adjusted to baseline characteristics (model 1) including age, gender, mechanical ventilation, APACHE II score, chronic co-morbidities (immunosuppressive disorders, heart failure, COPD, and medication- and insulin-dependent diabetes, elective surgery), nosocomial versus community acquired infection, bacteremia, Gram-negative organisms, fungal organisms, anaerobes (with Gram-positive organisms used as reference), pneumonia, urinary tract infection, primary bloodstream infection, catheter-related bloodstream infection, skin and soft tissue infection, creatinine clearance as calculated by MDRD and country. To adjust for the impact of potential changes in practice over time, we divided the study time period into four quartiles and we included the study period in the multivariate model. In the second model (model 2) we added the following variables related to sepsis interventions: inappropriate antimicrobial therapy, single versus combination antimicrobial therapy, delayed antimicrobial therapy > 3 hours, vasopressor doses (dopamine, norepinephrine, phenylephrine, epinephrine, and dobutamine), the use of a pulmonary artery catheter, activated protein C and the use of low-dose steroids. Among the variables included in the multivariate models, 3% of patients had missing values for APACHE II score and 3.5% of patients had missing data for MDRD, which were replaced by the means. Results were reported as odds ratios (ORs) with 95%CIs.

To study the association between BMI and the amount of fluid and antimicrobial dosage, we carried out multivariate linear regression analyses. We reported *P *values for the crude analysis and adjusted analysis for creatinine clearance. For the association of fluids and BMI, we also adjusted for pulmonary capillary wedge pressure in patients with a pulmonary artery catheter. A *P *value of < 0.05 was considered significant. SAS (SAS Institute, Cary, NC, USA) software was used for statistical analyses.

## Results

### Baseline characteristics

Of the 8,670 patients included during the study period, 2,882 (33.2%) had height and weight documented. When compared to excluded patients who did not have documented values of both weight and height in the medical records, there were no differences in age (*P *= 0.07), APACHE II score (*P *= 0.10) and use of mechanical ventilation (*P *= 0.29). However, excluded patients were more likely to have comorbid conditions (*P *= 0.007) and had lower hospital mortality compared to the included patients (*P *= 0.03).

Overall, 35.3% of the study population was in the normal weight group, 6.8% were underweight, 28.3% overweight, 23.6% obese and 5.4% very obese. Table 1 shows their baseline characteristics. Compared with the reference group (normal weight), very obese and underweight patients were younger. Obese and very obese patients were less likely to have immunosuppressive disorders and more likely to have heart failure and diabetes mellitus compared to those with normal BMI. With increasing BMI, admission heart rate, temperature and mean arterial pressure were higher and respiratory rate was lower. Creatinine was higher and platelet count lower with increasing BMI with the corresponding organ failure indicators following the same pattern (Table 1).

**Table 1:** Baseline characteristics among different groups of BMI

Variables	< 18.50 Number = 196	18.50 to 24.99 Number = 1,020	25.0 to 29.99 Number = 816	30.0 to -39.99 Number = 680	≥ 40 Number = 170	*P *value
Age (years), mean (SD)	59.1 (19.2)	62.2 (16.8)	63.5 (15.9)	62.2 (14.6)	58.4 (13.0)	< 0.001
Gender, female, number (%)	82 (41.8)	390 (38.2)	316 (38.7)	334 (49.1)	102 (60.0)	< 0.001
Actual body weight (kg), mean (SD)	47.3 (7.1)	63.4 (10.2)	77.3 (10.1)	93.4 (14.7)	128.9 (28.1)	< 0.001
Ideal body weight (kg), mean (SD)	61.7 (10.9)	62.9 (11.5)	62.4 (10.7)	60.4 (12.0)	58.3 (13.4)	< 0.001
Dosing body weight (kg), mean (SD)	58.1 (9.7)	63.0 (10.9)	66.1 (10.4)	68.6 (12.3)	75.9 (15.8)	< 0.001
Height (cms), mean (SD)	167.3 (10.6)	168.8 (11.1)	167.9 (10.1)	166.3 (11.5)	164.5 (13.3)	< 0.001
BMI (kg/m^2^), mean (SD)	16.8 (1.3)	22.2 (1.8)	27.3 (1.4)	33.6 (2.7)	47.4 (7.4)	< 0.001
Mechanical ventilation, number (%)	146 (74.5)	777 (76.2)	605 (74.1)	491 (72.2)	128 (75.3)	0.48
APACHE II, mean (SD)	25.5 (8.0)	25.7 (8.1)	25.6 (8.4)	25.4 (7.9)	24.4(7.3)	0.43
Admission physiologic data, mean (SD)						
Heart rate (beats/minute)	120.9 (30.3)	119.2 (29.4)	116.7 (29.9)	119.1 (29.5)	112.2 (31.0)	0.02
Respiratory rate (beats/minute)	29.0 (8.8)	27.0 (10.2)	26.6 (10.4)	26.0 (10.0)	25.8 (9.1)	0.005
Temperature (°C)	37.2 (1.8)	37.5 (1.7)	37.7 (1.7)	37.8 (1.6)	37.8 (1.7)	< 0.001
Mean arterial pressure (mmHg)	53.3 (13.3)	56.6 (16.5)	58.0 (19.0)	60.3 (20.1)	62.6 (22.2)	< 0.001
Co-morbidities, number (%)						
Immunosuppressive disorder	56 (28.6)	300 (29.4)	213 (26.1)	188 (27.7)	32 (18.8)	0.06
Liver failure	14 (7.1)	91 (8.9)	67 (8.2)	53 (7.8)	19 (11.2)	0.59
New York Heart Association class IV heart failure	16 (8.2)	70 (6.9)	95 (11.6)	77 (11.3)	21 (12.4)	0.002
Severe COPD (requiring medication or oxygen)	22 (11.2)	102 (10.0)	88 (10.8)	83 (12.2)	32 (18.8)	0.02
Chronic renal failure^a^	35 (17.9)	155 (15.2)	126 (15.4)	100 (14.7)	34 (20.0)	0.43
Chronic renal failure-dialysis dependence	17 (8.7)	81 (7.9)	45 (5.5)	41 (6.0)	12 (7.1)	0.20
Diabetes mellitus (medication dependent)	10 (5.1)	127 (12.5)	141 (17.3)	159 (23.4)	45 (26.5)	< 0.001
Diabetes mellitus (insulin-dependent)	11 (5.6)	76 (7.5)	82 (10.1)	80 (11.8)	35 (20.6)	< 0.001
Alcohol abuse	29 (14.8)	162 (15.9)	107 (13.1)	76 (11.2)	15 (8.8)	0.02
Elective surgery	28 (14.3)	160 (15.7)	159 (19.5)	130 (19.1)	25 (14.7)	0.09
Emergency surgery/trauma	17 (8.7)	97 (9.5)	73 (9.0)	49 (7.2)	8 (4.7)	0.19
No co-morbidity	58 (29.6)	285 (27.9)	227 (27.8)	170 (25.0)	38 (22.4)	0.33
Any co-morbidity	138 (70.4)	735 (72.1)	589 (72.2)	510 (75.0)	132 (77.6)	
Laboratory findings on day-1, mean (SD)						
White blood count (×10^9^/L)	16.1 (16.4)	16.1 (15.3)	16.5 (16.9)	17.9 (16.1)	16.0 (10.6)	0.20
Creatinine (μmol/L)	164.3 (148.2)	195.3 (160.6)	207.7 (167.9)	221.7 (165.2)	242.3 (201.8)	< 0.001
Bicarbonate (mmol/L)	19.4 (6.7)	19.1 (6.4)	18.8 (6.4)	19.0 (6.7)	20.1 (6.1)	0.32
Bilirubin (μmol/L)	33.2 (54.4)	41.5 (76.3)	44.1 (92.5)	41.5 (68.1)	31.1 (76.0)	0.29
Platelets (×10^9^/L)	206.9 (140.5)	198.4 (156.6)	185.6 (133.3)	210.3 (140.6)	223.5 (132.2)	0.004
International normalized ratio	1.8 (1.2)	1.7 (1.1)	1.8 (1.1)	1.8 (1.4)	1.7 (1.3)	0.78
Creatinine clearance (mL/minute), mean (SD)						
MDRD^b ^equation	85.9 (148.2)	56.5 (49.3)	48.1 (40.6)	43.1 (36.0)	37.1 (27.6)	< 0.001
Cockcroft-Gault equation	69.6 (98.6)	49.8 (41.3)	42.3 (34.3)	37.9 (29.9)	33.6 (22.7)	< 0.001
Source of infection, number (%)						
Community acquired infection	102 (52.0)	555 (54.4)	410 (50.3)	350 (51.5)	105 (61.8)	0.06
Nosocomial infection	94 (48.0)	465 (45.6)	406 (49.8)	330 (48.5)	65 (38.2)	
Bacteremia, Number (%)	68 (34.7)	361 (35.4)	289 (35.4)	266 (39.1)	48 (28.2)	0.10
Organ failures on day-1, number (%)						
Cardiovascular	196 (100)	1020 (100)	816 (100)	680 (100)	170 (100)	*
Renal	80 (40.8)	550 (53.9)	501 (61.4)	447 (65.7)	112 (65.9)	< 0.001
Respiratory	146 (74.5)	777 (76.2)	605 (74.1)	491 (72.2)	128 (75.3)	0.48
Hematologic	56 (28.6)	261 (25.6)	241 (29.5)	163 (24.0)	29 (17.1)	0.006
Metabolic	110 (56.1)	551 (54.0)	441 (54.0)	367 (54.0)	91 (53.5)	1.00
Central nervous system	47 (24.0)	289 (28.3)	210 (25.7)	168 (24.7)	45 (26.5)	0.44
Hepatic	32 (16.3)	195 (19.1)	157 (19.2)	144 (21.2)	31 (18.2)	0.60
Coagulation	89 (45.4)	454 (44.5)	350 (42.9)	284 (41.8)	69 (40.6)	0.70
Country, number (%)						
Canada	147 (75.0)	813 (79.7)	655 (80.3)	548 (80.6)	130 (76.5)	0.15
USA	28 (14.3)	101 (9.9)	90 (11.0)	75 (11.0)	28 (16.5)	
Saudi Arabia	21 (10.7)	106 (10.4)	71 (8.7)	57 (8.4)	12 (7.1)	
Period, number (%)						
≤ 1997	57 (29.1)	301 (29.5)	218 (26.7)	191 (28.1)	32 (18.8)	0.18
1998 to 2000	72 (36.7)	378 (37.1)	292 (35.8)	235 (34.6)	67 (39.4)	
2001 to 2002	57 (29.1)	296 (29.0)	265 (32.5)	207 (30.4)	62 (36.5)	
≥ 2003	10 (5.1)	45 (4.4)	41 (5.0)	47 (6.9)	9 (5.3)	

APACHE II, Acute Physiology and Chronic Health Evaluation II; BMI, body mass index;^a^Serum creatinine > 1.5 upper limit of normal,

^a^Serum creatinine > 1.5 upper limit of normal = Chronic renal failure

^b^MDRD: Modification of diet in renal disease

### Microbiologic data and sites of infections

Obese and very obese patients were more likely to have Gram-positive infections. Very obese patients were more likely to have sepsis from skin and soft tissue infections and less from pneumonia (Table 2). Table 3 shows the sepsis interventions and Table 4 shows the fluid and antibiotic dosage among the five groups of BMI.

**Table 2:** Microbiologic and sites of infection among different groups of BMI

Number (%)	< 18.50 Number = 196	18.50 to 24.99 Number = 1,020	25.0 to29.99 Number = 816	30.0 to 39.99 Number = 680	≥ 40 Number = 170	*P *value
Total culture negative	49 (25.0)	270 (26.5)	242 (29.7)	204 (30.0)	53 (31.2)	0.28
Total culture positive	147 (75.0)	750 (73.5)	574 (70.3)	476 (70.0)	117 (68.8)	
Gram-negative organisms	72 (36.7)	384 (37.7)	281 (34.4)	230 (33.8)	45 (26.5)	0.05
*Escherichia coli*	23 (11.7)	163 (16.0)	115 (14.1)	97 (14.3)	19 (1.2)	0.32
Klebsiella	16 (8.2)	62 (6.1)	41 (5.0)	36 (5.3)	7 (4.1)	0.38
*Pseudomonas aeruginosa*	13 (6.6)	68 (6.7)	48 (5.9)	28 (4.1)	8 (4.7)	0.23
Enterobacter species	5 (2.6)	27 (2.7)	21 (2.6)	25 (3.7)	1 (0.6)	0.25
*Haemophilus influenzae*	6 (3.1)	12 (1.2)	10 (1.2)	8 (1.2)	3 (1.8)	0.29
Acinetobacter species	4 (2.0)	10 (1.0)	6 (0.8)	11 (1.6)	2 (1.2)	0.38
Other Gram-negative bacilli	5 (2.6)	42 (4.1)	40 (4.9)	25 (3.7)	5 (2.9)	0.49
Gram-positive organisms	41 (20.9)	247 (24.2)	183 (22.4)	168 (24.7)	60 (35.3)	0.007
*Staphylococcus aureus*	19 (9.7)	110 (10.8)	81 (9.9)	71 (10.4)	22 (12.9)	0.81
*Streptococcus pneumonia*	9 (4.6)	60 (5.9)	37 (4.5)	37 (5.4)	8 (4.7)	0.74
*Streptococci faecalis*	4 (2.0)	19 (1.9)	17 (2.1)	17 (2.5)	6 (3.5)	0.68
*Streptococcus faecium*	2 (1.0)	11 (1.1)	8 (1.0)	4 (0.6)	3 (1.8)	0.69
Group A streptococcus species	2 (1.0)	18 (1.8)	17 (2.1)	14 (2.1)	10 (5.9)	0.01
Other β-hemolytic streptococci	3 (1.5)	12 (1.2)	10 (1.2)	13 (1.9)	8 (4.7)	0.01
Other Gram-positive bacilli	2 (1.0)	17 (1.7)	13 (1.6)	12 (1.8)	3 (1.8)	0.97
Fungi/Yeast	20 (10.2)	77 (7.6)	84 (10.3)	62 (9.1)	8 (4.7)	0.08
*Candida albicans*	11 (5.6)	44 (4.3)	50 (6.1)	37 (5.4)	7 (4.1)	0.46
Other candida/yeast	9 (4.6)	33 (3.2)	34 (4.2)	25 (3.7)	1 (0.6)	0.18
Anaerobes	10 (5.1)	26 (2.6)	17 (2.1)	14 (2.1)	4 (2.4)	0.15
Other organisms	4 (2.0)	16 (1.6)	9 (1.1)	2 (0.3)	0	0.04
Site of infection						
Lung	95 (48.5)	445 (43.6)	301 (36.9)	226 (33.2)	53 (31.2)	< 0.001
Pneumonia	91 (46.4)	435 (42.7)	296 (36.3)	223 (32.8)	51 (30.0)	0.45
Empyema	4 (2.0)	10 (1.0)	5 (0.6)	3 (0.4)	2 (1.2)	0.22
Intraabdominal	53 (27.0)	299 (29.3)	268 (32.8)	228 (33.5)	53 (31.2)	0.20
Intraabdominal abscess	5 (2.6)	23 (2.3)	23 (2.8)	28 (4.1)	8 (4.7)	0.15
Ascending Cholangitis	2 (1.0)	23 (2.3)	14 (1.7)	13 (1.9)	0	0.28
Cholecystitis	1 (0.5)	9 (0.9)	14 (1.7)	12 (1.8)	1 (0.6)	0.26
Ischemic bowel/bowel infarction	7 (3.6)	48 (4.7)	51 (6.3)	35 (5.2)	5 (2.9)	0.27
Viscous bowel perforation	21 (10.7)	104 (10.2)	104 (12.8)	66 (9.7)	20 (11.8)	0.34
Spontaneous bacterial peritonitis	3 (1.5)	31 (3.0)	14 (1.7)	17 (2.5)	1 (0.6)	0.16
*Clostridium difficile *enterocolitis/toxic megacolon	7 (3.6)	25 (2.5)	18 (2.2)	12 (1.8)	2 (1.2)	0.49
Infected pancreatic necrosis	0	4 (0.4)	11 (1.4)	16 (2.4)	3 (1.8)	0.002
Others	7 (3.6)	32 (3.1)	19 (2.3)	29 (4.3)	13 (7.7)	0.009
Skin and soft tissue	14 (7.1)	69 (6.8)	72 (8.8)	79 (11.6)	37 (21.8)	< 0.001
Cellulitis	2 (1.0)	7 (0.7)	11 (1.4)	19 (2.8)	12 (7.1)	< 0.001
Necrotizing soft tissue infections	5 (2.6)	20 (2.0)	16 (2.0)	23 (3.4)	12 (7.1)	0.001
Others	7 (3.6)	42 (4.1)	45 (5.5)	37 (5.4)	13 (7.7)	0.22
Genitourinary	17 (8.7)	92 (9.0)	80 (9.8)	70 (10.3)	18 (10.6)	0.88
Intravascular catheter infection	8 (4.1)	38 (3.7)	29 (3.6)	36 (5.3)	6 (3.5)	0.46
Primary bloodstream infection (bacteremia without identifiable source)	8 (4.1)	49 (4.8)	44 (5.4)	37 (5.4)	7 (4.1)	0.87
Systemically disseminated infection (including yeast and tuberculosis)	6 (3.1)	29 (2.8)	26 (3.2)	18 (2.7)	5 (2.9)	0.98
Septic arthritis	0	5 (0.5)	7 (0.9)	7 (1.0)	6 (3.5)	0.002

BMI, body mass index.

**Table 3:** Sepsis interventions related to septic shock among the five groups of BMI

Variable	< 18.50 Number = 196	18.50 to 24.99 Umber = 1,020	25.0 to 29.99 Number = 816	30.0 to 39.99 Number = 680	≥ 40 Number = 170	*P *value
Vasopressors used, Number (%)						
Dopamine	117 (59.7)	594 (58.2)	465 (57.0)	408 (60.0)	91 (53.5)	0.54
Norepinephrine	122 (62.2)	566 (55.5)	480 (58.8)	362 (53.2)	86 (50.6)	0.04
Phenylephrine	58 (29.6)	336 (32.9)	255 (31.3)	219 (32.2)	48 (28.2)	0.70
Epinephrine	12 (6.1)	45 (4.4)	41 (5.0)	31 (4.6)	15 (8.8)	0.15
Dobutamine	16 (8.2)	98 (9.6)	84 (10.3)	75 (11.0)	18 (10.6)	0.77
Vasopressor dose - at six hours, mean (SD)						
Dopamine (μcg/kg/minute)	7.7 (7.3)	6.7 (4.9)	6.5 (4.7)	6.4 (4.3)	6.2 (5.1)	0.22
Norepinephrine (μcg/kg/minute)	0.39 (0.41)	0.37 (0.60)	0.33 (0.43)	0.30 (0.47)	0.18 (0.18)	0.05
Phenylephrine (μcg/kg/minute)	3.4 (4.1)	2.2 (2.1)	2.1 (2.3)	2.0 (2.4)	1.2 (1.4)	0.003
Epinephrine (μcg/kg/minute)	0.85 (0.61)	0.30 (0.26)	0.33 (0.73)	0.18 (0.12)	0.21 (0.18)	0.04
Dobutamine (μcg/kg/minute)	5.1 (1.7)	4.4 (2.5)	4.7 (2.9)	4.0 (2.8)	4.4 (2.5)	0.82
Sepsis specific therapy						
Activated protein C, number (%)	9 (4.6)	36 (3.5)	29 (3.6)	42 (6.2)	13 (7.7)	0.01
Low-dose steroids, number (%)	73 (37.2)	305 (29.9)	256 (31.4)	222 (32.7)	46 (27.1)	0.19
Inappropriate antimicrobials therapy, number (%)	46 (23.5)	188 (18.4)	160 (19.6)	120 (17.7)	31 (18.2)	0.43
Single versus combined antimicrobial therapy, number (%)	142 (72.5)	711 (69.7)	568 (69.6)	456 (67.1)	118 (69.4)	0.62
Delay in antimicrobial therapy, mean (SD)	17.8 (30.3)	13.9 (24.0)	15.1 (26.0)	12.6 (18.8)	16.7 (29.9)	0.12
Others						
Pulmonary artery catheter, number (%)	91 (46.4)	513 (50.3)	428 (52.5)	386 (56.8)	89 (52.4)	0.04
Pulmonary capillary wedge pressure, (mmHG), mean (SD)	15.0 (5.2)	16.5 (6.1)	17.6 (6.1)	18.6 (6.4)	20.5 (6.7)	< 0.001
Cardiac output (L/minute), mean (SD)	6.4 (2.8)	7.1 (3.2)	7.6 (3.2)	7.7 (3.4)	8.7 (3.5)	< 0.001
Cardiac index(L/minute/m^2^), mean (SD)	4.1 (1.7)	4.1 (1.7)	4.0 (1.6)	3.8 (1.5)	3.9 (1.5)	0.11

BMI, body mass index.

**Table 4:** Fluid and antibiotic dosage among different groups of BMI

**Intervention**^**a**^ mean (SD)		< 18.50 Number = 196	18.50 to 4.99 Number = 1,020	25.0 to 29.99 Number = 816	30.0 to 39.99 Number = 680	≥ 40 Number = 170	*P *value	**Adjusted *P *value**^**b**^	Adjusted ***P *value**^**c**^
Crystalloid	Number = 1,506 (52.3%)	Number = 96 (49.0)	Number = 537 (52.6)	Number = 439 (53.8)	Number = 346 (50.9)	Number = 88 (51.8)			
	Total (ml)	2,580.3 (1,874.9)	2,690.0 (2,050.2)	2,861.7 (2,445.1)	2,537.4 (2,032.0)	2,602.3 (1,919.1)	0.30	0.76	0.59
	Dose per kg of actual body weight (ml/kg)	55.0 (40.1)	43.2 (33.4)	37.1 (30.8)	27.7 (22.0)	21.4 (16.8)	< 0.001	< 0.001	< 0.001
Colloid	Number = 1,506 (52.3%)	Number = 96 (49.0)	Number = 537 (52.6)	Number = 439 (53.8)	Number = 346 (50.9)	Number = 88 (51.8)			
	Total (ml)	656.8 (864.8)	509.8 (781.0)	593.5 (788.7)	705.5 (2,824.5)	538.9 (939.8)	0.44	0.79	0.31
	Dose per kg of actual body weight (ml/kg)	14.3 (19.3)	8.2 (12.7)	7.7 (10.2)	8.5 (44.2)	4.3 (7.2)	0.06	0.02	< 0.001
Packed red blood cell	Number = 94 (3.3%)	Number = 5 (2.6)	Number = 29 (2.8)	Number = 31(3.8)	Number = 24 (3.5)	Number = 5 (2.9)			
	Total (ml)	462.0 (180.7)	601.0 (630.3)	522.5 (243.2)	502.0 (250.0)	961.2 (781.5)	0.26	0.68	0.79
	Dose per kg of actual body weight (ml/kg)	9.6 (4.1)	9.7 (10.8)	6.8 (3.1)	5.4 (3.0)	7.3 (5.5)	0.18	0.02	0.53
Fresh frozen plasma	Number 70 (2.4%)	Number = 4 (2.0)	Number = 22 (2.2)	Number = 21(2.6)	Number = 18 (2.6)	Number = 5 (2.9)			
	Total (ml)	737.5 (715.7)	498.7 (224.4)	600.0 (336.9)	526.8 (333.1)	1,260.0 (1,34.9)	0.01	0.005	0.02
	Dose per kg of actual body weight (ml/kg)	18.9 (20.8)	8.2 (4.4)	7.8 (4.7)	5.8 (3.7)	10.4 (8.4)	0.01	0.77	0.62
Piperacillin/tazo-bactam	Number = 564 (19.6%)	Number = 46 (23.5)	Number = 214 (21.0)	Number = 145 (17.8)	Number = 129 (19.0)	Number = 30 (17.6)			
	Total dose (mg)	10,214.7 (3,216.3)	10,714.8 (3,155.2)	11,005.3 (3,074.6)	10,754.8 (3,264.7)	10,766.7 (2,699.9)	0.68	0.06	
	Dose per kg of dosing body weight	183.5 (66.7)	174.8 (59.9)	168.3 (56.1)	161.0 (59.9)	133.2 (40.2)	0.001	< 0.001	
Cefotaxime	Number = 772 (26.8%)	Number = 51 (26.0)	Number = 297 (29.1)	Number = 212 (26.0)	Number = 173 (25.4)	Number = 39 (22.9)			
	Total dose (mg)	3,549.0 (1,932.0)	3,477.8 (1,821.8)	3,787.7 (1,857.4)	3,359.8 (1,531.0)	4,256.4 (2,220.9)	0.02	0.006	
	Dose per kg of dosing body weight	62.1 (38.5)	56.5 (31.6)	57.2 (28.7)	49.7 (24.4)	53.5 (30.2)	0.04	0.32	
Imipenem	Number = 242 (8.4%)	Number = 15 (7.7)	Number = 79 (7.7)	Number = 75 (9.2)	Number = 56 (8.2)	Number = 17 (10.0)			
	Total dose (mg)	1,566.7 (457.7)	1,665.2 (597.5)	1,693.3 (700.6)	1,705.4 (796.5)	1,426.5 (584.7)	0.60	0.36	
	Dose per kg of dosing body weight	25.9 (9.0)	27.0 (9.9)	24.9 (10.3)	24.2 (13.1)	7.3 (54.6)	0.002	< 0.001	
Gentamycin	Number = 511 (17.7%)	Number = 33 (16.8)	Number = 184 (18.0)	Number = 149 (18.3)	Number = 122 (17.9)	Number = 23 (13.5)			
	Total dose (mg)	173.3 (82.4)	219.9 (241.4)	256.7 (229.4)	239.4 (118.3)	262.0 (121.9)	0.18	0.01	
	Dose per kg of dosing body weight	3.2 (1.5)	3.6 (4.0)	3.8 (3.2)	3.3 (1.7)	3.2 (1.6)	0.63	0.96	
Meropenem	Number = 277 (9.6%)	Number = 19 (9.7)	Number = 83 (8.1)	Number = 85 (10.4)	Number = 70 (10.3)	Number = 20 (11.8)			
	Total dose (mg)	2,342.1 (1,179.1)	1,807.2 (791.8)	1,865.9 (990.6)	1,678.6 (920.9)	2,050.0 (916.2)	0.07	0.75	
	Dose per kg of dosing body weight	40.7 (19.4)	29.8 (15.0)	28.8 (15.7)	25.0 (15.5)	26.1 (11.3)	0.003	0.01	
Levofloxacin	Number = 301 (10.4%)	Number = 28 (14.3)	Number = 110 (10.8)	Number = 81 (9.9)	Number = 62 (9.1)	Number = 20 (11.8)			
	Total dose (mg)	426.8 (118.2)	489.8 (164.3)	488.0 (205.8)	441.5 (251.8)	462.5 (91.6)	0.32	0.82	
	Dose per kg of dosing body weight	8.0 (2.5)	8.0 (3.1)	7.1 (2.8)	6.2 (4.4)	5.3 (1.3)	< 0.001	< 0.001	
Vancomycin	Number = 771 (26.8%)	Number = 53 (27.0)	Number = 261(25.6)	Number = 209 (25.6)	Number = 194 (28.5)	Number = 54 (32.1)			
	Total dose (mg)	1,292.5 (521.6)	1,421.2 (630.3)	1,380.4 (578.4)	1,353.1 (623.0)	1,441.4 (680.6)	0.54	0.20	
	Dose per kg of dosing body weight	28.5 (13.7)	22.9 (11.2)	17.9 (7.6)	14.8 (7.2)	11.3 (5.8)	< 0.001	< 0.001	
Ciprofloxacin	Number = 634 (22.0%)	Number = 39 (19.9)	Number = 218 (21.4)	Number = 186 (22.8)	Number = 165 (24.3)	Number = 26 (15.3)			
	Total dose (mg)	682.9 (280.3)	674.4 (237.0)	671.4 (255.8)	670.3 (363.1)	682.7 (210.2)	1.0	0.13	
	Dose per kg of dosing body weight	13.0 (5.2)	10.9 (4.4)	10.1 (4.1)	9.1 (5.0)	7.6 (2.6)	< 0.001	< 0.001	
Ampicillin	Number = 340 (11.8%)	Number = 19 (9.7)	Number = 122 (12.0)	Number = 115 (14.1)	Number = 70 (10.3)	Number = 14 (8.2)			
	Total dose (mg)	4,106.6 (1,788.6)	4,002.3 (1,940.8)	4,326.1 (2,019.7)	4,072.1 (1,64.0)	5,071.4 (2,302.7)	0.31	0.01	
	Dose per kg of dosing body weight	71.8 (38.4)	64.8 (32.2)	66.5 (33.0)	61.3 (31.0)	67.0 (36.3)	0.74	0.85	
Amphotericin B	Number = 102 (3.5%)	Number = 5 (2.6)	Number = 34 (3.3)	Number = 36 (0.4)	Number = 25 (3.7)	Number = 2 (1.2)			
	Total dose (mg)	3,897.2 (4,330.8)	5,531.1 (3,998.5)	6,866.2 (5,977.6)	5,079.2 (4,933.2)	10,520.0 (10,521.7)	0.35	0.53	
	Dose per kg of dosing body weight	85.4 (91.9)	84.9 (59.7)	90.9 (80.2)	54.3 (52.4)	83.5 (86.7)	0.33	0.15	
Ceftazidime	Number = 292 (10.1%)	Number = 25 (1.3)	Number = 106 (10.4)	Number = 89 (10.9)	Number = 58 (8.5)	Number = 14 (8.2)			
	Total dose (mg)	2,710.0 (1,154.0)	3,547.2 (1,867.7)	2,995.3 (1,691.5)	3,025.9 (1,590.6)	3,285.7 (1,683.8)	0.08	0.10	
	Dose per kg of dosing body weight	47.2 (18.3)	57.5 (30.7)	46.9 (28.0)	43.9 (23.6)	42.7 (17.7)	0.01	0.48	
Ceftriaxone	Number = 232 (8.0%)	Number = 12 (6.1)	Number = 77 (7.5)	Number = 67 (7.6)	Number = 58 (8.5)	Number = 18 (10.6)			
	Total dose (mg)	1,666.7 (492.4)	1,655.8 (1,052.1)	1,776.1 (884.6)	1,672.4 (943.8)	1,611.1 (607.7)	0.93	0.77	
	Dose per kg of dosing body weight	31.9 (10.2)	26.1 (14.4)	27.5 (12.7)	24.6 (13.7)	21.5 (8.7)	0.20	0.02	
Cefuroxime	Number = 772 (26.8%)	Number = 19 (9.7)	Number = 74 (7.3)	Number = 68 (8.3)	Number = 55 (8.1)	Number = 11(6.5)			
	Total dose (mg)	2,578.9 (920.6)	2,171.8 (1,063.2)	2,216.9 (808.0)	2,386.4 (842.7)	2,181.8 (975.3)	0.39	0.68	
	Dose per kg of dosing body weight	45.3 (18.9)	35.1 (17.0)	32.7 (12.9)	34.6 (15.0)	26.7 (12.1)	0.01	0.02	

**^a^**Crystalloid, colloid, crystalloid equivalent volume, red blood cell and fresh frozen plasma for first six hours. All others for day 1. ^b^Adjusted for creatinine clearance. ^c^Adjusted for creatinine clearance and pulmonary capillary wedge pressure. BMI, body mass index.

### Outcomes

**Figure 1:**
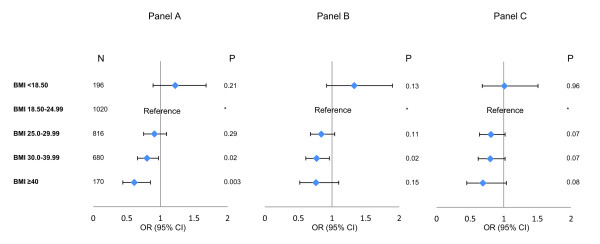
**Odds ratios and confidence intervals of hospital mortality among different BMI groups in multivariate logistic regression analysis**. **Panel A**: Crude analysis. **Panel B**: Adjusted analysis to baseline characteristics (model 1). **Panel C**: Adjusted analysis to baseline characteristics and sepsis interventions (model 2). BMI, body mass index.

Crude hospital mortality, ICU mortality, hospital LOS and ICU LOS differed among the BMI groups (Table 5). Compared with patients with normal BMI, the crude OR for obese patients was 0.80, 95% CI 0.66 to 0.97 and for the morbidly obese was 0.61, 95% CI 0.44 to 0.85. After adjustment for baseline characteristics (model 1) and for baseline characteristics and sepsis interventions (model 2), the OR and CI became non-significant statistically (Figure 1).

**Table 5:** Outcomes among different groups of BMI

	< 18.50 Number = 196	18.50 to 24.99 Number = 1,020	25.0 to 29.99 Number = 816	30.0 to 39.99 Number = 680	≥ 40 Number = 170	*P *value
Hospital mortality, number (%)	121 (61.7)	580 (56.9)	444 (54.4)	349 (51.3)	76 (44.7)	0.003
ICU mortality, number (%)	102 (52.0)	451 (44.2)	335 (41.1)	265 (39.0)	57 (33.5)	0.001
ICU length of stay, mean (SD)	9.9 (9.4)	10.5 (12.5)	11.4 (14.7)	11.2 (14.3)	12.2 (12.7)	0.30
Hospital length of stay, mean (SD)	25.1 (33.6)	25.1 (31.8)	27.8 (35.2)	26.5 (34.8)	34.3 (44.2)	0.02

BMI, body mass index.

## Discussion

Our study found that: 1) the BMI of patients with septic shock varies considerably; 2) septic shock in the obese and very obese differs in the presentation, sites of infection and microbiology compared to non-obese patients; 3) sepsis interventions, especially the volume of resuscitation fluids and doses of antibiotics, do not appear to take into consideration the variations in BMI, leading to considerable differences in weight-adjusted doses; and 4) crude hospital mortality of obese and morbidly obese patients with septic shock was lower than normal weight patients, but this was explained at least in part by differences in baseline characteristics and sepsis interventions.

Our study revealed that obese and morbidly patients with septic shock display some differences in their clinical presentation, site and type of infection. Indeed, underweight and very obese patients with septic shock had fewer hemodynamic disturbances and, thus, required lower doses of vasopressors (expressed as mcg/kg/minute for norepinephrine and epinephrine) than normal weight patients, although with comparable APACHE II scores. Further, the source of infection in obese patients was more likely to be related to skin and soft tissue infections and less likely to be related to pneumonia with predominantly Gram-positive microorganisms.

Our study examined the influence of weight and BMI on sepsis interventions. We observed that similar volumes of resuscitation fluids and similar total doses of antimicrobials were administered regardless of BMI. This translated to lower volume/kg and dose/kg in the obese and very obese patients compared to the normal BMI patients even after adjustment for wedge pressure and creatinine clearance. For example, we found that very obese patients received 27.7 ± 22.0 ml/kg crystalloid volume in the first six hours compared to 55.0 ± 40.1 ml/kg in the underweight group. This probably reflects routine clinical practice which is not weight-based as physicians often administer a standard bolus of 1,000 ml crystalloid or 300 to 500 ml colloid, irrespective of body weight. Unlike pediatricians, physicians caring for adult patients do not routinely take weight and height into consideration in decision-making. The issue is further confounded by the large number of correction formulae for weight adjustment (actual, adjusted, ideal, predicted, antimicrobial dosing body weights) that are mostly not based on clinical outcome studies, leaving the bedside practitioner unclear about the best approach [6,29]. Additionally, it remains unclear whether dosing vasopressors, such as norepinephrine and epinephrine, based on weight (as mcg/kg/minute) is superior to non-weight based dosing (as mcg/minute) as both approaches are seen in practice and in clinical trials [30,31].

Our study shows that crude hospital mortality of obese and very obese patients with septic shock was lower than normal weight patients. Because the effect became insignificant after adjustment, we believe that the observed differences in mortality were explained partially by baseline characteristics and sepsis interventions. The effect of obesity on outcome of critically ill patients has been a subject of considerable debate [32]. Two meta-analyses revealed discordant results, with one showing no difference in mortality between obese and normal-weight critically ill adults [33] and another demonstrating a trend toward decreased risk of mortality in overweight and obese patients compared with those with normal BMI [11]. In septic shock, a retrospective study of 301 patients with septic shock found that overweight and obese patients had lower mortality than those with normal weight [18].

Despite this supportive evidence, how a condition that increases cause-specific mortality, such as ischemic heart disease, diabetes, cancer and respiratory diseases, can be neutral or even provides a survival advantage during critical illness, remains perplexing. Several possible explanations exist. First, epidemiologic and statistical reasons may explain the association. The lower observed mortality might be related to lead-time bias and to the earlier age in the very obese septic patients. Additionally, the obese and morbidly obese patients may be 'healthier' than the normal BMI patients. Obese patients displayed more diabetes and related complications, while the normal weight had more devastating illnesses, including hematologic and solid organ malignancies. Therefore, the observed differences in outcome may be related to worse outcome in the normal weight patients and not to 'better' outcome in the obese. Additionally, septic shock in the obese and very obese may be less severe as shown by the lower vasopressor doses. The calibration of APACHE II in morbidly obese patients has to be validated, because several of its components, such as oxygenation, may be altered due to obesity and not to the acute illness. Finally, although we adjusted for several co-morbidities, residual confounding cannot be ruled out.

Second, our study identified differences in sepsis interventions as one of the potential explanations for this apparent paradox. Several studies have highlighted disparities in care provided to patients in the obese and very obese groups compared with patients in other categories [34,35]. At present, there is no strong evidence to support whether a weight-based approach for fluid resuscitation, antibiotic or vasopressor dosing is superior to the non-weight-based approach. Our study shows clearly that obese patients received a lower amount of resuscitation fluids and dose of antibiotics based on weight. Although one cannot establish a causal relationship with mortality, emerging evidence suggests that over-resuscitation may have detrimental effects [36,37]. Therefore, it is appealing to speculate that this may have contributed to the higher mortality in both lower and normal BMI patients, thus making obesity appear neutral or protective. Further, our study did not examine other important sepsis interventions, such as tidal volume [38,39] and doses of other medications, all of which deserve further study.

Third, the observed association of obesity with lower mortality may be related to true biological reasons. One explanation is an underlying difference in metabolic and immune response to acute illness [40]. Obesity activates many inflammatory pathways that deregulate physiological responses, which maintain metabolic homeostasis including insulin and leptin sensitivity. One can speculate that a superimposed episode of acute inflammation triggered by sepsis on top of the chronic inflammation may induce a different immune response than in naïve patients. A second possible explanation has been variations in vital signs leading to perception of higher severity of illness [8]. However, in our cohort of patients with septic shock, we found that very obese patients had a slightly lower heart rate and respiratory rate and higher blood pressure and temperature, which may be related to a different response but could also be simply related to less severe shock in contrast to what has been suggested by Martino *et al. *[8].

It is important to note that these findings apply to the individual episode of septic shock only. Once septic shock sets in, our data suggest that patients in the obese and very obese groups may have outcomes that are similar or not worse than those in other groups. We did not capture data about the association between obesity and predisposition to sepsis in the general population. However, considering the younger age of very obese patients in the septic population, one may question whether obesity actually increases the risk for septic shock. This point and the life-long consequences of septic shock on the obese patients need to be studied in a population-based cohort.

Our study should be interpreted in light of its strengths and limitations. To our knowledge, this is the largest study to date specifically addressing the impact of obesity on outcomes among patients with septic shock. The inclusion of patients from 28 ICUs based in three geographic regions lends the study wide generalizability. In terms of limitations, the findings only highlight associations and cause-effect relationships cannot be inferred. We used the weight and height as documented in the medical records, the precision of which could not be verified. The availability of BMI data on only one third of patients may have led to selection bias. However, the comparison between excluded and included patients showed minor differences that are unlikely to explain the magnitude of the observed differences among BMI groups. Furthermore, this lack of data may represent perception bias as healthcare workers are more likely to measure height and weight in extreme groups of BMI. Our study did not address the possibility of measurement bias arising from systematic errors in measuring weight, height and blood pressure across different BMI groups. We adjusted for severity of illness as measured by APACHE II and other possible confounders; we cannot rule out residual confounding especially considering the large number of possible factors that may influence outcome in acutely ill patients. Finally, we do not have data on the sagittal abdominal diameter, which has been shown to be an independent risk factor of death in critically ill patients [41].

## Conclusions

In the septic shock population, we observed the obesity paradox (lower mortality in the obese) reported in other populations. This may be related in part to differences in patient characteristics. However, the true paradox may lie in the variations in the sepsis interventions, such as the administration of resuscitation fluids and antimicrobial therapy. Further studies are warranted to examine whether a weight-based approach to common therapeutic interventions in septic shock influences outcome.

## Key messages

• The presentation, microbiology and organ failures are different among obese patients compared to the non-obese.

• Very obese patients presented at an early age to the ICU with septic shock and had more underlying chronic co-morbidities compared to non-obese patients.

• There is a considerable variation in the volume of fluid resuscitation per kg and antibiotic dosing among different BMI groups.

• The obesity paradox (lower mortality in the obese) as reported in other populations was observed in septic shock patients as well.

• The true paradox may lie in the variations in the sepsis interventions, such as the administration of resuscitation fluids.

## Abbreviations

ANOVA: analysis of variance; APACHE: Acute Physiology and Chronic Health Evaluation; BMI: body mass index; CATSS: Cooperative Antimicrobial Therapy of Septic Shock; COPD: chronic obstructive pulmonary disease; LOS: length of stay; MDRD: modification of diet in renal disease; OR: odds ratio; SAS: statistical analysis software; WHO: World Health Organization.

## Competing interests

The authors declare that they have no competing interests.

## Authors' contributions

YMA had full access to all of the data in the study and takes responsibility for the integrity and the accuracy of the data analysis YMA participated in conception and design, in analysis and interpretation of data, drafted the manuscript, critically revised the manuscript for important intellectual content and approved the final version to be published. SID participated in analysis and interpretation of data, helped to draft the manuscript, critically revised the manuscript for important intellectual content and approved the final version to be published. HMT performed statistical analysis, critically revised the manuscript for important intellectual content and approved the final version to be published. AHR collected the data for study, participated in statistical analysis, critically revised the manuscript for important intellectual content and approved the final version to be published. ARB, MKK, DF, JEP, KEW, SK, SZ, GM, ASK and AK participated in conception, design and development of the database, helped in analysis and interpretation of data, helped in drafting of the manuscript, critically revised the manuscript for important intellectual content and approved the final version to be published.

## Supplementary Material

Additional file 1 **Appendix A: Definitions of organ failures**. Appendix B: Formulas. Click here for file

## References

[B1] Katzmarzyk PT Reeder BA Elliott S Joffres MR Pahwa P Raine KD Kirkland SA Paradis G Body mass index and risk of cardiovascular disease, cancer and all-cause mortality Can J Public Health 2012 103 147 151 22530540 10.1007/BF03404221 PMC6974265

[B2] Yusuf S Hawken S Ounpuu S Bautista L Franzosi MG Commerford P Lang CC Rumboldt Z Onen CL Lisheng L Tanomsup S Wangai P Jr Razak F Sharma AM Anand SS INTERHEART Study Investigators Obesity and the risk of myocardial infarction in 27,000 participants from 52 countries: a case-control study Lancet 2005 366 1640 1649 10.1016/S0140-6736(05)67663-5 16271645

[B3] Hossain P Kawar B El Nahas M Obesity and diabetes in the developing world--a growing challenge N Engl J Med 2007 356 213 215 10.1056/NEJMp068177 17229948

[B4] Al-Baghli NA Al-Ghamdi AJ Al-Turki KA El-Zubaier AG Al-Ameer MM Al-Baghli FA Overweight and obesity in the eastern province of Saudi Arabia Saudi Med J 2008 29 1319 1325 18813420

[B5] Institute of Medicine of the National Academics Recommendation on Obseity Prevention [http://iom.edu/~/media/Files/Report%20Files/2012/APOP/APOP_rb.pdf] Accessed on 14 May 2009

[B6] El-Solh AA Clinical approach to the critically ill, morbidly obese patient Am J Respir Crit Care Med 2004 169 557 561 10.1164/rccm.200309-1256CC 14982823

[B7] Sakr Y Madl C Filipescu D Moreno R Groeneveld J Artigas A Reinhart K Vincent JL Obesity is associated with increased morbidity but not mortality in critically ill patients Intensive Care Med 2008 34 1999 2009 10.1007/s00134-008-1243-0 18670756

[B8] Martino JL Stapleton RD Wang M Day AG Cahill NE Dixon AE Suratt BT Heyland DK Extreme obesity and outcomes in critically ill patients Chest 2011 140 1198 1206 10.1378/chest.10-3023 21816911 PMC3205847

[B9] Hutagalung R Marques J Kobylka K Zeidan M Kabisch B Brunkhorst F Reinhart K Sakr Y The obesity paradox in surgical intensive care unit patients Intensive Care Med 2011 37 1793 1799 10.1007/s00134-011-2321-2 21818652

[B10] Habbu A Lakkis NM Dokainish H The obesity paradox: fact or fiction? Am J Cardiol 2006 98 944 948 10.1016/j.amjcard.2006.04.039 16996880

[B11] Oliveros H Villamor E Obesity and mortality in critically ill adults: a systematic review and meta-analysis Obesity (Silver Spring) 2008 16 515 521 10.1038/oby.2007.102 18239602

[B12] Akinnusi ME Pineda LA El Solh AA Effect of obesity on intensive care morbidity and mortality: a meta-analysis Crit Care Med 2008 36 151 158 10.1097/01.CCM.0000297885.60037.6E 18007266

[B13] Angus DC Linde-Zwirble WT Lidicker J Clermont G Carcillo J Pinsky MR Epidemiology of severe sepsis in the United States: analysis of incidence, outcome, and associated costs of care Crit Care Med 2001 29 1303 1310 10.1097/00003246-200107000-00002 11445675

[B14] Dombrovskiy VY Martin AA Sunderram J Paz HL Rapid increase in hospitalization and mortality rates for severe sepsis in the United States: a trend analysis from 1993 to 2003 Crit Care Med 2007 35 1244 1250 10.1097/01.CCM.0000261890.41311.E9 17414736

[B15] Martin GS Mannino DM Eaton S Moss M The epidemiology of sepsis in the United States from 1979 through 2000 N Engl J Med 2003 348 1546 1554 10.1056/NEJMoa022139 12700374

[B16] Adrie C Alberti C Chaix-Couturier C Azoulay E De Lassence A Cohen Y Meshaka P Cheval C Thuong M Troche G Garrouste-Orgeas M Timsit JF Epidemiology and economic evaluation of severe sepsis in France: age, severity, infection site, and place of acquisition (community, hospital, or intensive care unit) as determinants of workload and cost J Crit Care 2005 20 46 58 10.1016/j.jcrc.2004.10.005 16015516

[B17] Vachharajani V Russell JM Scott KL Conrad S Stokes KY Tallam L Hall J Granger DN Obesity exacerbates sepsis-induced inflammation and microvascular dysfunction in mouse brain Microcirculation 2005 12 183 194 15828130 10.1080/10739680590904982

[B18] Wurzinger B Dunser MW Wohlmuth C Deutinger MC Ulmer H Torgersen C Schmittinger CA Grander W Hasibeder WR The association between body-mass index and patient outcome in septic shock: a retrospective cohort study Wien Klin Wochenschr 2010 122 31 36 20177857 10.1007/s00508-009-1241-4

[B19] Bone RC Balk RA Cerra FB Dellinger RP Fein AM Knaus WA Schein RM Sibbald WJ Definitions for sepsis and organ failure and guidelines for the use of innovative therapies in sepsis. The ACCP/SCCM Consensus Conference Committee. American College of Chest Physicians/Society of Critical Care Medicine Chest 1992 101 1644 1655 10.1378/chest.101.6.1644 1303622

[B20] Kumar A Ellis P Arabi Y Roberts D Light B Parrillo JE Dodek P Wood G Simon D Peters C Ahsan M Chateau D Cooperative Antimicrobial Therapy of Septic Shock Database Research Group Initiation of inappropriate antimicrobial therapy results in a fivefold reduction of survival in human septic shock Chest 2009 136 1237 1248 10.1378/chest.09-0087 19696123

[B21] Kumar A Zarychanski R Light B Parrillo J Maki D Simon D Laporta D Lapinsky S Ellis P Mirzanejad Y Martinka G Keenan S Wood G Arabi Y Feinstein D Kumar A Dodek P Kravetsky L Doucette S Cooperative Antimicrobial Therapy of Septic Shock (CATSS) Database Research Group Early combination antibiotic therapy yields improved survival compared with monotherapy in septic shock: a propensity-matched analysis Crit Care Med 2010 38 1773 1785 10.1097/CCM.0b013e3181eb3ccd 20639750

[B22] WHO classification of body mass index (BMI) [http://apps.who.int/bmi/index.jsp?introPage=intro_3.html] Accessed on 9 April 2012

[B23] Knaus WA Draper EA Wagner DP Zimmerman JE APACHE II: a severity of disease classification system Crit Care Med 1985 13 818 829 10.1097/00003246-198510000-00009 3928249

[B24] Levey AS Bosch JP Lewis JB Greene T Rogers N Roth D A more accurate method to estimate glomerular filtration rate from serum creatinine: a new prediction equation. Modification of Diet in Renal Disease Study Group Ann Intern Med 1999 130 461 470 10.7326/0003-4819-130-6-199903160-00002 10075613

[B25] Cockcroft DW Gault MH Prediction of creatinine clearance from serum creatinine Nephron 1976 16 31 41 10.1159/000180580 1244564

[B26] Michels WM Grootendorst DC Verduijn M Elliott EG Dekker FW Krediet RT Performance of the Cockcroft-Gault, MDRD, and new CKD-EPI formulas in relation to GFR, age, and body size Clin J Am Soc Nephrol 2010 5 1003 1009 10.2215/CJN.06870909 20299365 PMC2879308

[B27] Wurtz R Itokazu G Rodvold K Antimicrobial dosing in obese patients Clin Infect Dis 1997 25 112 118 10.1086/514505 9243045

[B28] Kumar A Roberts D Wood KE Light B Parrillo JE Sharma S Suppes R Feinstein D Zanotti S Taiberg L Gurka D Kumar A Cheang M Duration of hypotension before initiation of effective antimicrobial therapy is the critical determinant of survival in human septic shock Crit Care Med 2006 34 1589 1596 10.1097/01.CCM.0000217961.75225.E9 16625125

[B29] Erstad BL Dosing of medications in morbidly obese patients in the intensive care unit setting Intensive Care Med 2004 30 18 32 10.1007/s00134-003-2059-6 14625670

[B30] Annane D Vignon P Renault A Bollaert PE Charpentier C Martin C Troche G Ricard JD Nitenberg G Papazian L Azoulay E Bellissant E CATS Study Group Norepinephrine plus dobutamine versus epinephrine alone for management of septic shock: a randomised trial Lancet 2007 370 676 684 10.1016/S0140-6736(07)61344-0 17720019

[B31] Russell JA Walley KR Singer J Gordon AC Hebert PC Cooper DJ Holmes CL Mehta S Granton JT Storms MM Cook DJ Presneill JJ Ayers D VASST Investigators Vasopressin versus norepinephrine infusion in patients with septic shock N Engl J Med 2008 358 877 887 10.1056/NEJMoa067373 18305265

[B32] O'Brien JM Jr Welsh CH Fish RH Ancukiewicz M Kramer AM Excess body weight is not independently associated with outcome in mechanically ventilated patients with acute lung injury Ann Intern Med 2004 140 338 345 10.7326/0003-4819-140-5-200403020-00009 14996675

[B33] Hogue CW Jr Stearns JD Colantuoni E Robinson KA Stierer T Mitter N Pronovost PJ Needham DM The impact of obesity on outcomes after critical illness: a meta-analysis Intensive Care Med 2009 35 1152 1170 10.1007/s00134-009-1424-5 19189078

[B34] O'Brien JM Jr Philips GS Ali NA Aberegg SK Marsh CB Lemeshow S The association between body mass index, processes of care, and outcomes from mechanical ventilation: a prospective cohort study Crit Care Med 2012 40 1456 1463 10.1097/CCM.0b013e31823e9a80 22430246

[B35] O'Brien JM Jr Phillips GS Ali NA Lucarelli M Marsh CB Lemeshow S Body mass index is independently associated with hospital mortality in mechanically ventilated adults with acute lung injury Crit Care Med 2006 34 738 744 16521268 10.1097/01.CCM.0000202207.87891.FC PMC1868702

[B36] Wiedemann HP Wheeler AP Bernard GR Thompson BT Hayden D deBoisblanc B Connors AF Jr Hite RD Harabin AL Comparison of two fluid-management strategies in acute lung injury N Engl J Med 2006 354 2564 2575 16714767 10.1056/NEJMoa062200

[B37] Maitland K Kiguli S Opoka RO Engoru C Olupot-Olupot P Akech SO Nyeko R Mtove G Reyburn H Lang T Brent B Evans JA Tibenderana JK Crawley J Russell EC Levin M Babiker AG Gibb DM FEAST Trial Group Mortality after fluid bolus in African children with severe infection N Engl J Med 2011 364 2483 2495 10.1056/NEJMoa1101549 21615299

[B38] Anzueto A Frutos-Vivar F Esteban A Bensalami N Marks D Raymondos K Apezteguia C Arabi Y Hurtado J Gonzalez M Tomicic V Abroug F Elizalde J Cakar N Pelosi P Ferguson ND Ventila group Influence of body mass index on outcome of the mechanically ventilated patients Thorax 2011 66 66 73 10.1136/thx.2010.145086 20980246

[B39] Gong MN Bajwa EK Thompson BT Christiani DC Body mass index is associated with the development of acute respiratory distress syndrome Thorax 2010 65 44 50 10.1136/thx.2009.117572 19770169 PMC3090260

[B40] Stapleton RD Dixon AE Parsons PE Ware LB Suratt BT The association between BMI and plasma cytokine levels in patients with acute lung injury Chest 2010 138 568 577 10.1378/chest.10-0014 20435656 PMC2940070

[B41] Paolini JB Mancini J Genestal M Gonzalez H McKay RE Samii K Fourcade OA Predictive value of abdominal obesity vs. body mass index for determining risk of intensive care unit mortality Crit Care Med 2010 38 1308 1314 20228682 10.1097/CCM.0b013e3181d8cd8b

