# Supplementary material for: Clinical characteristics, sepsis interventions and outcomes in the obese patients with septic shock: an international multicenter cohort study
Source: Crit Care. 2013 Apr 17;17(2):R72. doi: 10.1186/cc12680 (PMC3672731; doi:10.1186/cc12680)
Supplement: Additional file 1 — Appendix A: Definitions of organ failures. Appendix B: Formulas. [file cc12680-S1.DOC]

**Appendix A**

**Definitions of organ failures**

1. **Cardiovascular System Dysfunction**

- SBP ≤90 mm Hg or drop of more than 40 mm from normal systolic pressure

Or

- MAP ≤65 mm Hg for at least one hour despite adequate fluid resuscitation (2 L saline equivalent)

Or

- Need for vasopressors.

1. **Renal Dysfunction**

- Elevation of normal baseline serum creatinine to >1.5 X normal value.

1. **Respiratory System Dysfunction**

- Ventilation required (more than immediate for surgery).

1. **Hematologic Dysfunction**

- Platelet count <80,000/mm.

1. **Metabolic Dysfunction**

- Lactate level >3 mmol/L (1.5 times the upper limit of the normal).

1. **Central Nervous System Dysfunction**

- An acute alteration in mental status not attributable to sedation.

1. **Hepatic Dysfunction**

- Elevation of normal baseline serum total bilirubin to ≥2 mg/dl or 35 µmol/L (unless due to primary biliary disease e.g. ascending cholangitis, Cholecystitis).

**Appendix B**

**Formulas**

1. **Creatinine clearance**

1. ***Cockcroft* and *Gault equation***[***^1^***](#_ENREF_1)

CrCl = [(140 - age) x ideal body weight (IBW)] / (Scr x 72) (x 0.85 for females).

1. **Modification of Diet in Renal Disease *(MDRD)***[***^2^***](#_ENREF_2)

GFR = 186 x (SCR)^-1.154^ x (age in years)^-0.203^ x 1.210 (if patient is black) x 0.742 (if female).

1. **Ideal body weight (IBW)**[**^3^**](#_ENREF_3)

For males, IBW (kg) = 50 + (0.91 x (height in cm - 152.4))

For females, IBW (kg) = 45.5 + (0.91 x (height in cm - 152.4))

1. **Dosing body weight (DBW)**[**^4^**](#_ENREF_4)

DBW= IBW + 0.25 (actual body weight - IBW)

**Table: Dose adjustments for antimicrobial therapy in very obese patients**

| **Antibiotics** | **Recommended dosing weight** | **References** |
| --- | --- | --- |
| β-lactam | IBW + 0.3 (ABW-IBW) | [^5^](#_ENREF_5) |
| Vancomycin | Actual body weight | [^6^](#_ENREF_6) |
| Ciprofloxacin | IBW + 0.45 (ABW-IBW) | [^7^](#_ENREF_7) |
| Gentamycin | IBW + 0.43 (ABW-IBW) | [^8^](#_ENREF_8) |
| Amikacin | IBW + 0.38 (ABW-IBW) | [^9^](#_ENREF_9) |
| Amphotericin | Actual body weight | [^10^](#_ENREF_10) |

*IBW= Ideal body weight; ABW = Actual body weight*

*CrCl: Creatinine clearance; Scr: Serum creatinine*

**References**

1. Cockcroft DW, Gault MH. Prediction of creatinine clearance from serum creatinine. *Nephron.* 1976;16(1):31-41.

2. Levey AS, Bosch JP, Lewis JB, Greene T, Rogers N, Roth D. A more accurate method to estimate glomerular filtration rate from serum creatinine: a new prediction equation. Modification of Diet in Renal Disease Study Group. *Ann Intern Med.* Mar 16 1999;130(6):461-470.

3. Ventilation with lower tidal volumes as compared with traditional tidal volumes for acute lung injury and the acute respiratory distress syndrome. The Acute Respiratory Distress Syndrome Network. *N Engl J Med.* May 4 2000;342(18):1301-1308.

4. Cutts ME, Dowdy RP, Ellersieck MR, Edes TE. Predicting energy needs in ventilator-dependent critically ill patients: effect of adjusting weight for edema or adiposity. *Am J Clin Nutr.* Nov 1997;66(5):1250-1256.

5. Wurtz R, Itokazu G, Rodvold K. Antimicrobial dosing in obese patients. *Clin Infect Dis.* Jul 1997;25(1):112-118.

6. Blouin RA, Bauer LA, Miller DD, Record KE, Griffen WO, Jr. Vancomycin pharmacokinetics in normal and morbidly obese subjects. *Antimicrob Agents Chemother.* Apr 1982;21(4):575-580.

7. Allard S, Kinzig M, Boivin G, Sorgel F, LeBel M. Intravenous ciprofloxacin disposition in obesity. *Clin Pharmacol Ther.* Oct 1993;54(4):368-373.

8. Traynor AM, Nafziger AN, Bertino JS, Jr. Aminoglycoside dosing weight correction factors for patients of various body sizes. *Antimicrob Agents Chemother.* Feb 1995;39(2):545-548.

9. Bauer LA, Blouin RA, Griffen WO, Jr., Record KE, Bell RM. Amikacin pharmacokinetics in morbidly obese patients. *Am J Hosp Pharm.* Apr 1980;37(4):519-522.

10. Darovic GO, Vanriper S. Arterial pressure monitoring. In: Darovic GO, editor.Hemodynamic monitoring: invasive and noninvasive clinical application, 2nd ed. Philadelphi, PA: WB Saunders Company; 1995, p. 177-210.
